# Structure of the PLP-Form of the Human Kynurenine Aminotransferase II in a Novel Spacegroup at 1.83 Å Resolution

**DOI:** 10.3390/ijms17040446

**Published:** 2016-03-25

**Authors:** Alireza Nematollahi, Guanchen Sun, Stephen J. Harrop, Jane R. Hanrahan, W. Bret Church

**Affiliations:** 1 Group in Biomolecular Structure and Informatics, Faculty of Pharmacy, University of Sydney, Sydney, NSW 2006, Australia; anem7250@uni.sydney.edu.au (A.N.); gsun2866@uni.sydney.edu.au (G.S.); 2 MX Beamlines, Australian Synchrotron, 800 Blackburn Road, Clayton, VIC 3168, Australia; stephen.harrop@synchrotron.org.au; 3 Faculty of Pharmacy, University of Sydney, Sydney, NSW 2006, Australia; jane.hanrahan@sydney.edu.au

**Keywords:** kynurenine aminotransferase, crystal structure, neurodegenerative diseases, pyridoxal phosphate (PLP)

## Abstract

Kynurenine aminotransferase II (KAT-II) is a 47 kDa pyridoxal phosphate (PLP)-dependent enzyme, active as a homodimer, which catalyses the transamination of the amino acids kynurenine (KYN) and 3-hydroxykynurenine (3-HK) in the tryptophan pathway, and is responsible for producing metabolites that lead to kynurenic acid (KYNA), which is implicated in several neurological diseases such as schizophrenia. In order to fully describe the role of KAT-II in the pathobiology of schizophrenia and other brain disorders, the crystal structure of full-length PLP-form hKAT-II was determined at 1.83 Å resolution, the highest available. The electron density of the active site reveals an aldimine linkage between PLP and Lys263, as well as the active site residues, which characterize the fold-type I PLP-dependent enzymes.

## 1. Introduction

Kynurenic acid (KYNA), with several specific biological activities, is known to antagonize all ionotropic glutamate receptors. The antagonisation of the *N*-methyl-d-aspartate (NMDA) receptor occurs by binding at the glycine binding site [1] and at the α-amino-3-hydroxy-5-methyl-4-isoxazolepropionic acid receptor (AMPA) as well as at the lesser studied Kainate receptor, and at each of which then causes neuronal excitation [2,3], and accelerates the neuronal death rate [4]. Therefore the physiological role described for KYNA which prevents glutamate receptors from becoming excessively excited establishes it as crucial for brain stability under normal physiological conditions [5]. In addition, it is clear that increased KYNA levels protects neurons against ischemic brain damage, and also anti-seizure activity is known [6,7]. More recently, studies revealed that α7-nicotinic acetylcholine receptors (α7-nAChRs) are inhibited by KYNA, and this results in a decreased release of glutamate, which balances levels of extracellular dopamine [8]. Furthermore, among other literature reports effects on the G protein-coupled receptor 35 (GPR35) are attributed to KYNA, a receptor which is mainly expressed in immune cells [9], suggesting KYNA might play a more general role in immunological regulation. As inflammation (resulting from viral and bacterial infections) can activate the tryptophan catabolic pathway [10,11], the ensuing tryptophan catabolism decreases plasma levels of tryptophan and increases KYNA plasma levels [12,13]. KYNA also exhibits antioxidant activity due to its capability of scavenging free radical agents [14,15] which is independent of its pharmacological actions at receptors.

Regardless of the evidence for the participation of KYNA in neuroprotection, the potential for cognitive damage in the central nervous system (CNS) which results from the involvement of glutamatergic and cholinergic neurotransmission needs appropriate consideration [16], especially in the context of the negative symptoms of schizophrenia. Studies on the cerebrospinal fluid (CFS) of patients affected by schizophrenia revealed that KYNA levels were raised [17,18]. Consequently, KYNA can potentially have both the neuroprotective and the cognitive impairing effects. As a result of the latter observations several neurodegenerative diseases are linked with the kynurenine metabolic pathway and brain KYNA levels. In addition to cognitive impairment brain KYNA levels have been shown to play a key role in other CNS diseases such as Huntington’s disease [19], Alzheimer’s disease and schizophrenia [20].

It is believed that in the CNS of mammals kynurenine aminotransferase (KAT) isozymes are responsible for the synthesis of KYNA [21]. Both KAT I and KAT II are seen to be substantially expressed in astrocytes [22], and are actively involved in the human brain which has the requirement for KYNA [23,24]. Our focus here is on KAT-II, which is a biologically active homodimer, and reportedly produces almost 75% of KYNA in human brain [25]. More recently several researchers have downplayed the importance of KAT I and KAT III in the brain [26]. The KATs irreversibly convert l-kynurenine (l-KYN), the main intermediate of the kynurenine pathway into KYNA, an endogenous neuroactive agent in CNS [25], as part of the main route of the tryptophan degradation pathway [27].

KAT-II consists of 425 amino acid residues with its catalytic site at the homodimeric interface. It belongs to the subgroup I_β_ and 1_γ_ of the pyridoxal-5′-phosphate (PLP) dependent aminotransferases [28,29] with its optimum activity at pH 7.0–7.5. PLP acts as a coenzyme and is covalently linked to Lys263 by a “Schiff base” reaction, resulting in an aldimine linkage. As the initial step of the enzymatic reaction pathway, the substrate L-KYN binds at the active site of the kynurenine aminotransferase and consequently the aldimine bond is broken, and the PLP then binds to the α-amino group of the l-KYN. In the ensuing steps this α-amino group is transferred to the coenzyme, converting the PLP to the pyridoxamine phosphate (PMP) form. Regeneration of PLP is achieved by the transfer of the α-amino group to the α-keto acid, leaving the α-keto acid co-substrate with l-KYN, which spontaneously undergoes a ring closure producing KYNA [30]. In this paper we describe the three-dimensional (3D) crystal structure of the PLP form of hKAT-II (PDB entry: 5EUN) in the novel space group *P*4_3_2_1_2, at 1.83 Å resolution, which is the highest available for the hKAT-II structure. The high resolution facilitates further study of the active site role including nearby residues and their possible involvement in the catalysis mechanism. Our structure is also valuable for the rational design of inhibitors for the purposes of their consideration as therapeutics for numerous CNS diseases in humans.

## 2. Results and Discussion

### 2.1. Purification and Activity Assay of hKAT-II

**Figure 1:**
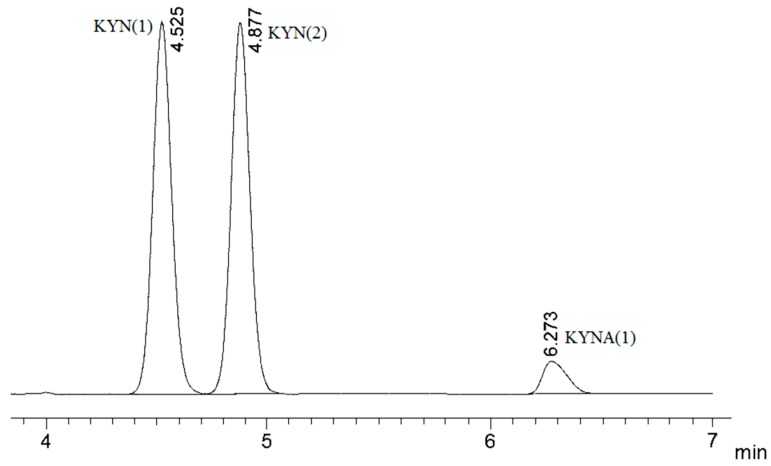
HPLC-based activity assay in which the mobile phase used was a water-acetonitrile (93:7) mixture and the detection wavelength was 330 nm. Injection (1) hKAT-II incubated with kynurenine (KYN), pyridoxal phosphate (PLP) and α-ketoglutarate in which kynurenic acid (KYNA) peak was observed; Injection (2) indicates the control that was missing hKAT-II in otherwise the same reaction mixture only has detected the KYN peak.

Using the Ni-NTA column chromatography method, the recombinantly expressed hKAT-II enzyme was purified, and its activity with the substrate (KYN) and co-substrate (α-ketoglutarate) was determined using HPLC (Figure 1). The activity assay results were consistent with previous reports of recombinant hKAT-II activity with α-ketoglutarate as co-substrate (Figure 1) [31,32].

### 2.2. Overall Structure

**Figure 2:**
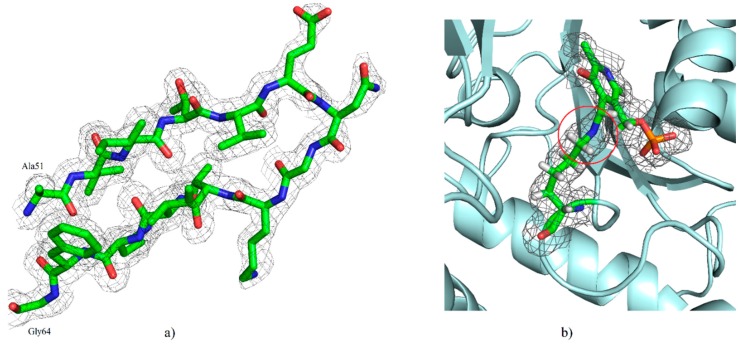
(**a**) Illustrative electron-density depiction from the final 2Fo–Fc map (grey at the 1.0 σ level), highlighting the agreement of antiparallel β-sheets (β1–β2; between Ala51 and Gly64) with the maps; (**b**) The 2Fo-Fc electron density map (grey at the 0.5 σ level) of lysine-pyridoxal-5′-phosphate (LLP)263, in which the aldimine linkage is highlighted by a red circle.

**Figure 3:**
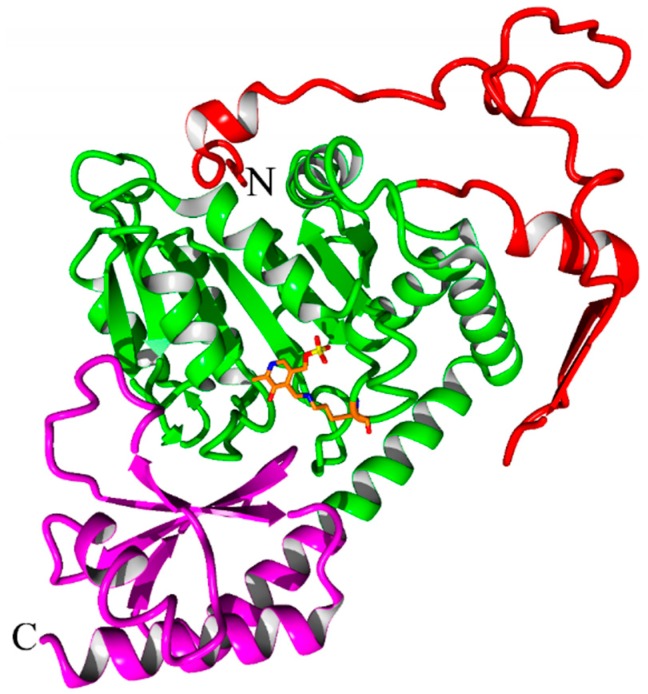
The structure of hKAT-II. The secondary structure elements are shown with the cofactor, PLP. The N-terminal arm is depicted in red, the large domain in green, the C-terminal arm is in magenta and the LLP263 is shown in orange stick representation. The termini are labelled with N and C.

**Figure 4:**
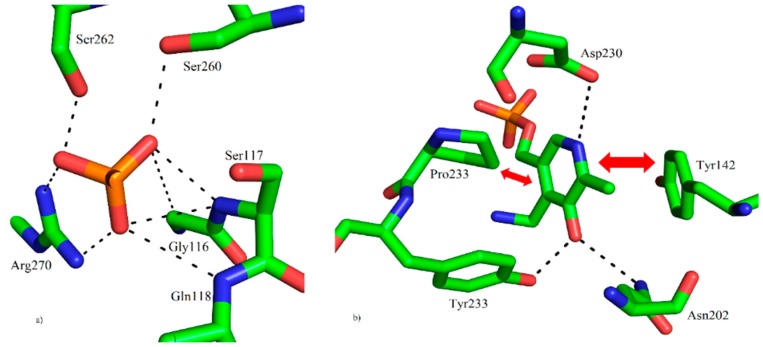
(**a**) The binding cup interactions of the phosphate group of LLP in the resting state of hKAT-II. The phosphate of LLP forms polar interactions with the residues Gly116, Ser117, Gln118, Ser262 and Arg-270; (**b**) Hydrophilic (hydrogen bond) interactions of Tyr233 and Asn202 with the pyridoxal ring of LLP, as well as polar interactions of Asp230 with pyridine nitrogen of LLP, which is a characteristic of the fold type-I of the PLP-dependent enzyme family. The hydrophobic interactions are with the sidechains of Tyr142 and Pro233, highlighted by red arrows. Hydrogen bonds are all represented with black broken lines.

The crystal of our hKAT-II holoenzyme, with the PLP form of the co-factor, diffracted to 1.83 Å. The crystals belonged to the novel tetragonal space group *P*4_3_2_1_2 with unit cell dimensions of *a* = 102.46 Å, *b* = 102.46 Å and *c* = 86.24 Å (α, β and γ = 90°). The asymmetric unit contains one molecule with an estimated solvent content of 48.1%. The molecular replacement (MR) solution had a log likelihood gain (LLG) of 12999 and a translation function Z-score of 107. The final fully refined structure was achieved with a final *R*-free and *R*-work of 19.89% and 17.30%, respectively, in which the agreement between them indicates over-fitting is unlikely. The final model contains all the residues of a total of 425, containing the one modified residue, LLP (lysine-pyridoxal-5′-phosphate (LLP), which is substituted for Lys263), and 438 solvent molecules. The geometry and stereochemistry of the structure were assessed by MolProbity [33] and 95.5% of residues are within the most favored regions and other 4.5% are within the allowed regions of the Ramachandran plot. Two *cis* peptide conformations were determined, at Pro140 and 203. The hKAT-II structure contains 37% α-helix, 15% β-sheet, 12% turn, 35% coil and 1% 3_10_ helix. Unambiguous electron density was generally observed, and the N-terminal arm consisting of an antiparallel β-sheet (β1–β2 strands; between Ala51 and Gly64) (Figure 2a). The state in which the side chain amino group of Lys263 forms an aldimine bond with PLP, also referred to as the resting state is the state of the hKAT-II observed with no ambiguity in the electron density maps. (Figure 2b). Also the N-terminal arm is observed rolled into the location away from the active site, which does not otherwise affect the active site pocket. The large domain of the protein consists of seven β strands (β3–β9) and the small domain (also known as the C-terminal arm) consists of three β-sheets (β10–β12) (Figure 3). From previous studies [34,35] of the PLP-dependent enzymes of fold type I the binding cup is seen to include hydrogen bonds and salt bridges, which involves the phosphate group of LLP as anticipated (shown in Figure 4a). The oxygen of the pyridine ring hydroxyl moiety of LLP interacts with Asn202 and Tyr233, and the pyridine ring is also sitting parallel to and alongside the Tyr142 ring. Additionally, the pyridoxal ring of LLP has a hydrophobic interaction with Pro232, not previously reported in the other available aminotransferase structures [4]. The side chain carboxylate oxygen of Asp230 takes part in a hydrogen bond with the NH pyridinium of LLP, which is seen to exist across all fold-type I proteins of the PLP-dependent enzyme family, which is generally considered crucial for enzyme function [36,37,38] (Figure 4b). Our hKAT-II structure crystal has minimal crystallographic complexity as there is only one molecule in the asymmetric unit, compared to other available structures which have been reported with up to 4 crystallographically independent molecules (*i.e.*, PDB entry 2R2N, at 1.95 Å resolution). The PLP ligand linked through the aldimine bond to Lys263, required for activity in the PLP-dependent enzyme families, is not always observed as examples exist in the apo form without the PLP, as in the case of the structures reported by Ku *et al.*, 2006 [39] (PDB entry 2C44) and Hasse *et al.*, 2013 [40] (PDB entry 4LGL). The biological dimer is observed faithfully in the crystal structure. To assess the experimental precision in the determination of the atomic positions in a protein crystal structure, the diffraction precision index (DPI) is the metric to which one defers [41]. DPI for all ligand free hKAT-II entries were calculated based on the formula using several variables pertinent to the precision calculations and summarized in Table 1, showing the superior precision of atomic positions determined in our structural studies.

## 3. Materials and Methods

### 3.1. Enzyme Expression and Purification

Using the online Genscript tool followed by manual intervention the hKAT-II gene was optimized for heterologous expression in E. coli and the nucleotide sequence was constructed by Genscript Corporation, Piscatway, NJ, USA. The optimized sequence was sub-cloned into a pET15b vector via NdeI/BamHI restriction sites. The expression vector was transformed into Rosetta 2 competent cells, the protein expression induced by adding 0.1 mM isopropyl β-d-1-thiogalactopyranoside (IPTG) for 24 h at 15 °C, and later bacterial pellets were harvested by centrifugation and stored at −80 °C. The pET15b vector added a hexa-histidine (6 × His) tag to the N-terminus of the expressed protein which allowed it to be purified using nickel-nitrilotriacetic acid (Ni-NTA) column chromatography. The purified recombinant hKAT-II was concentrated to 16 mg/mL using an Amicon^®^ ultra 4 mL centrifugal filter unit with a membrane size of 30 kDa. The enzyme purity was evaluated by SDS-PAGE analysis [32]. Enzyme production is summarized in Table 2.

### 3.2. Activity Assay

The activity assay was designed based on the previously described method with slight modifications [4]. A 50 µL reaction mixture containing 5 mM of l-kynurenine, 5 mM of α-ketoglutarate, 40 µM PLP and 0.9 µg purified hKAT2 in 10 mM phosphate buffer (pH 7.5) was used to determine the activity of the recombinant protein. The reaction mixture was incubated at 37 °C for 10 min and then an equal volume of 0.8 M formic acid was added to terminate the reaction. After centrifuging at 11,000× *g* at 4 °C for 10 min the supernatant was stored for the HPLC assay. KYN and KYNA peaks were detected at 330 nm using a C-18 reverse-phase column with water-acetonitrile (93:7) *v*/*v* % as the mobile phase.

### 3.3. Crystallization, Data Collection, Structure Solution and Refinement

Crystals of the recombinant human kynurenine aminotransferase II were grown using the vapor diffusion method with hanging drops technique. Protein (1 μL) at a concentration of 7 mg/mL was mixed with an equal volume of a reservoir solution containing 200 mM NaCl, 0.1 M NaCitrate pH 5.6, 24% PEG4K and equilibrated against 1 mL of a reservoir solution at 20 °C (as described in Table 3). Tetragonal bipyramidal crystals grew to a maximum dimension of 0.3 × 0.4 × 0.8 mm in one week.

Diffraction data were collected at the Australian Synchrotron Beamline MX2 (Clayton, Australia) at 100 K using the Blue-Ice software package [43]. Data processing was done using the XDS program with Aimless scaling and merging [44,45]. The structure of recombinant human kynurenine aminotransferase II was determined by molecular replacement (MR) using PHASER [46] with an ensemble structure generated by using the SCULPTOR [47] and ENSEMBLER features implemented in PHENIX (Ver 1.10.1-2155) [48] from the PDB entries (2R2N, 2QLR, 2VGZ and 3DC1) [4,49,50,51,52,53] and homology model built by YASARA [54]. The built model was refined by Phenix.refine [55], and to improve the quality of the protein model and perform corrections, modifications of the side-chain χ dihedral angles were performed with the side-chain rotator tool in KiNG (Kinemage) version 2.21 [56] with the most recent version of the “Penultimate Rotamer Library” [57]. Additionally the Backrub tool [58] implemented in KiNG (Kinemage) was used to adjust the protein backbone to apply fixes in the cases which were not improved by side chain movement alone. The rebuilding process was accomplished using the amended model and then the coordinates, individual temperaturefactors and occupancies were refined and the weight of geometry was optimized against the data. The final structure was refined at 1.83 Å resolution and the quality of the structure was evaluated by PHENIX comprehensive validation tools [33,59]. Data collection, processing and structure refinement statistics are summarized in Table 4. Structure figures were created using PyMOL [60] and YASARA [54]. Coordinates and structure factors have been deposited at the Protein Data Bank (PDB) server with PDB entry 5EUN.

### 3.4. Homology Modeling

In order to obtain the high-accuracy ensemble structure as a reference model for molecular replacement, the homology modeling technique was used. The homology model of hKAT-II was constructed using YASARA [54]. The primary sequence of hKAT-II was obtained from the Swiss-Prot database (Q8N5Z0) [61] which has a total of 425 residues in the monomer. Using PSI-BLAST 209 hits were found, and 25 models were built using the highest score templates. Specifically, first an alignment of target and template residues was calculated for each of the templates, and the sequence identity and the sequence similarity determined. The alignment considers structural information, as well as the predicted target secondary structure [62]. The loops were then modelled and optimized by conformation sampling and optimization of all the sidechains with a medium computational demand. Optimization occurred using electrostatic and knowledge-based packing interactions, with consideration of solvation effects as implemented in the YASARA2 force field, achieving fine tuning of the model. Finally, YASARA provides a hybrid model by combining the best parts of the 25 constructed models, in an attempt to increase the accuracy beyond each of the contributory individual models. The hybrid model with a z-score of −0.101 was selected as the best model, and it was of satisfactory quality to provide success in the molecular replacement procedure.

## 4. Conclusions

Human kynurenine aminotransferase II makes the largest contribution to KYNA production in human brains and plays a key role in several CNS disorders. Therefore, it is a biological target of interest that may be important in considerations of how to overcome neurodegenerative diseases. In this paper the native structure of hKAT-II (PLP form) at 1.83 Å has been determined and refined, providing the highest resolution structure reported. This resultant model gives a complete, detailed description of the binding site of hKAT II, which is of considerable practical use, especially in the context of the challenge to design novel therapeutic lead inhibitors targeting this enzyme.

## Figures and Tables

**Table 1:** The diffraction precision index (DPI) of hKAT-II PDB entries.

PDB Entry Code	DPI * (Å)
5EUN (structure reported here)	0.12
2R2N	0.20
2QLR	0.50
2VGZ	0.31
3DC1	0.32

* Calculated DPI (Å) using *R*_work_ using Webserver Program [42].

**Table 2:** Enzyme production.

Source Organism	Homo Sapiens
DNA source	Synthesized (GenScript, Piscataway, NJ, USA)
5′ sequence	*BamHI/NdeI* site: GGATCCCATATG
3′ sequence	Stop codon and *BamHI* site: TAATAAGGATCC
Cloning vector	pUC57
Expression vector	pET15b
Expression host	*E. coli* Rosetta 2

**Table 3:** Crystallization.

Crystallization Method	Hanging Drop Vapor Diffusion
Plate type	24-well Tissue plate
Temperature (K)	293.15
Protein concentration (mg/mL)	7
Buffer of protein solution	20 mM Tris-HCl, pH 8.0, 50 mM NaCl
Reservoir solution	200 mM NaCl, 0.1 M, NaCitrate pH 5.6, 24% PEG4K
Volume and ratio of drop	2 μL, 1:1 ratio
Volume of reservoir (mL)	1

**Table 4:** Data collection and refinement statistics.

Diffraction Source	Australian Synchrotron MX2
Wavelength (Å)	0.9537
Temperature (K)	100
Detector	ADSC QUANTUM 315r CCD
Crystal-to-detector distance (mm)	250
Rotation range per image (°)	1.0
Total rotation range (°)	180
Exposure time per image (s)	1
Space group	*P4_3_2_1_2*
Unit cell (Å, °)	*a* = *b* = 102.46, *c* = 86.24 (α, β and γ = 90)
Resolution (Å)	39.74–1.825 (1.891–1.825) *
Total reflections	81,444 (6884)
Unique reflections	40,739 (3147)
Multiplicity	2.0 (2.0)
Completeness (%)	98.5 (85.1)
*〈I/*σ*(I)〉*	17.64 (2.87)
Wilson *B*-factor	17.44
*R*_merge_	0.0231 (0.1859)
*R*_meas_	0.03267 (0.2629)
CC_1/2_	0.999 (0.909)
Reflections used in refinement	39644 (3147)
Reflections used for *R*_free_	1942 (152)
*R*_work_	0.1730 (0.2148)
*R*_free_	0.1988 (0.2696)
CC_work_	0.958 (0.906)
CC_free_	0.932 (0.780)
Protein residues	425
R.m.s. (bonds)	0.004
R.m.s (angles)	0.72
Average *B*-factor	27.95
Ramachandran favored (%)	95.5
Ramachandran allowed (%)	4.5
Ramachandran outliers (%)	0
Rotamer outliers (%)	0.81
Clashscore	2.69
PDB ID	5EUN

* Statistics for the highest-resolution shell are shown in parentheses.

## Abbreviations

KAT-II	Kynurenine aminotransferase II (KAT-II)
KYNA	Kynurenic acid
NMDA	*N*-methyl-d-aspartate
AMPA	α-amino-3-hydroxy-5-methyl-4-isoxazolepropionic acid
α7-nAChRs	α7-nicotinic acetylcholine receptors
CNS	Central nervous system
L-KYN	l-kynurenine
PLP	Pyridoxal-5′-phosphate
6xHis	Hexa-histidine
Ni-NTA	Nickel-nitrilotriacetic acid
IPTG	Isopropyl β-d-1-thiogalactopyranoside
DPI	Diffraction precision index
